# CSN6 deregulation impairs genome integrity in a COP1-dependent pathway

**DOI:** 10.18632/oncotarget.3151

**Published:** 2015-01-31

**Authors:** Hyun Ho Choi, Chun-Hui Su, Lekun Fang, Jin Zhang, Sai-Ching J. Yeung, Mong-Hong Lee

**Affiliations:** ^1^ Department of Molecular and Cellular Oncology, The University of Texas MD Anderson Cancer Center, Houston, TX 77030, USA; ^2^ Institute of Biosciences and Technology, Texas A&M University Health Science Center, Houston, TX 77030, USA; ^3^ Key Laboratory of Breast Cancer Prevention and Therapy, Tianjin Medical University, Ministry of Education, Tianjin Medical University Cancer Institute and Hospital, National Clinical Research Center of Cancer, Tianjin, People's Republic of China; ^4^ Department of Emergency Medicine, The University of Texas MD Anderson Cancer Center, Houston, TX 77030, USA; ^5^ Departiment of Endocrine Neoplasia and Hormonal Disorders, The University of Texas MD Anderson Cancer Center, Houston, TX 77030, USA; ^6^ Program in Cancer Biology, The University of Texas Graduate School of Biomedical Sciences at Houston, Houston, TX 77030, USA; ^7^ Program in Genes and Development, The University of Texas Graduate School of Biomedical Sciences at Houston, Houston, TX 77030, USA

**Keywords:** p27, COP1, 14-3-3σ, ubiquitination, DNA damage

## Abstract

Understanding genome integrity and DNA damage response are critical to cancer treatment. In this study, we identify CSN6's biological function in regulating genome integrity. Constitutive photomorphogenic 1 (COP1), an E3 ubiquitin ligase regulated by CSN6, is downregulated by DNA damage, but the biological consequences of this phenomenon are poorly understood. p27^Kip1^ is a critical CDK inhibitor involved in cell cycle regulation, but its response to DNA damage remains unclear. Here, we report that p27^Kip1^ levels are elevated after DNA damage, with concurrent reduction of COP1 levels. Mechanistic studies showed that during DNA damage response COP1's function as an E3 ligase of p27 is compromised, thereby reducing the ubiquitin-mediated degradation of p27^Kip1^. Also, COP1 overexpression leads to downregulation of p27^Kip1^, thereby promoting the expression of mitotic kinase Aurora A. Overexpression of Aurora A correlates with poor survival. These findings provide new insight into CSN6-COP1-p27^Kip1^-Aurora A axis in DNA damage repair and tumorigenesis.

## INTRODUCTION

The COP9 signalosome (CSN) is a protein complex involved in protein degradation, transcriptional activation [1, 2], signal transduction [3–6], and tumorigenesis [5, 7–10]. The contribution of the CSN's subunits in cancer has not been well elucidated. Mammalian CSN subunits are involved in developmental processes: targeted disruptions of mammalian *Csn2, Csn3, Csn5*, and *Csn8* resulted in defective embryo development [11–14]. In our previous study, we performed targeted disruption of the *Csn6* gene in mice and found that *Csn*6−/− mice developed until 7.5 days post-coitus but not beyond this time [9]. Also, *Csn6*+/− mouse tumor experiments showed that *Csn6* haplo-insufficiency helps impede the development of cancer [9], suggesting that CSN6 signaling regulation is critical for tumor development. However, the mechanism and biological consequence of CSN6 overexpression in cancer remain unclear.

COP1 E3 ubiquitin ligase contains RING-finger, a coiled-coil, and WD40-repeat domains. COP1 is a crucial mediator to block photomorphogenesis in the dark through the ubiquitinated proteasomal degradation of light-induced transcription factor HY5 [15]. Mammalian COP1 regulates various cellular functions, such as proliferation and survival, by facilitating the degradation of physiological substrates through ubiquitin-mediated protein degradation [16, 17]. Many of the ubiquitinated targets of COP1 are involved in tumorigenesis, including p53 and 14-3-3σ tumor suppressors [6, 18], c-JUN [19], transducer of regulated CREB activity 2 (TORC2, a glucose metabolite regulator) [20], FOXO1 [21], and nucleosome remodeling factor MTA1 [22]. Also, we show that COP1 is a downstream target of CSN6 [6]. COP1 itself is autoubiquitinated, and this ubiquitination process is regulated by COP9 signalosome subunit 6 (CSN6) [6], a protein involved in Cullin neddylation [10]. COP1 is phosphorylated by ATM on S387 following DNA damage [23], which results in COP1 nuclear exclusion-mediated by 14-3-3σ and subsequent p53 activation [24, 25]. However, it is not clear how COP1 further involves in DNA damage response.

The tumor suppressor p27 is critical for regulating the cell cycle transition from the G0/G1 to the S phase [26–28]. Levels of p27 are tightly regulated to control cell cycle progression. p27 is a critical CDK inhibitor that can negatively regulate cell cycle progression. Cell responds to DNA damage with a cell cycle arrest for further DNA repair [29, 30], but there is a knowledge gap regarding detailed regulation of CDK inhibitors during DNA damage. DNA damage induces p53 accumulation, which in turn induces 14-3-3σ or p21 expression to execute cell cycle arrest [31–34]. It is not clear whether p27^Kip1^ (abbreviated as p27) [26, 29] is regulated in this process. p27 levels are mainly regulated through polyubiquitination. The ubiquitin ligase component F-box protein Skp2 regulates polyubiquitination of p27 and mediates its degradation [35–38]. However, in the absence of Skp2, p27 is still degraded, suggesting that other E3 ubiquitin ligases may regulate p27 turnover [39]. PirH2, a RING containing protein [40], is another identified E3 ligases for p27 [39]. It remains to be characterized if any other E3 ligase may regulate p27.

Here, we found that CSN6 is involved in chromosomal integrity. The downstream target of the CSN6 axis—COP1 is in this process. COP1 coordinates with p27 and Aurora A expression to regulate genome integrity and DNA damage repair. Our studies characterize the signaling of the COP1-p27-Aurora A axis in DNA damage response. These results provide insight into the process of DNA damage by defining a new mechanism for posttranslational regulation of p27. Our findings also implicate a specific mechanism by which p27 is deregulated in human cancers.

## RESULTS

### CSN6 expression leads to mitotic defect and ROS production

**Figure 1:**
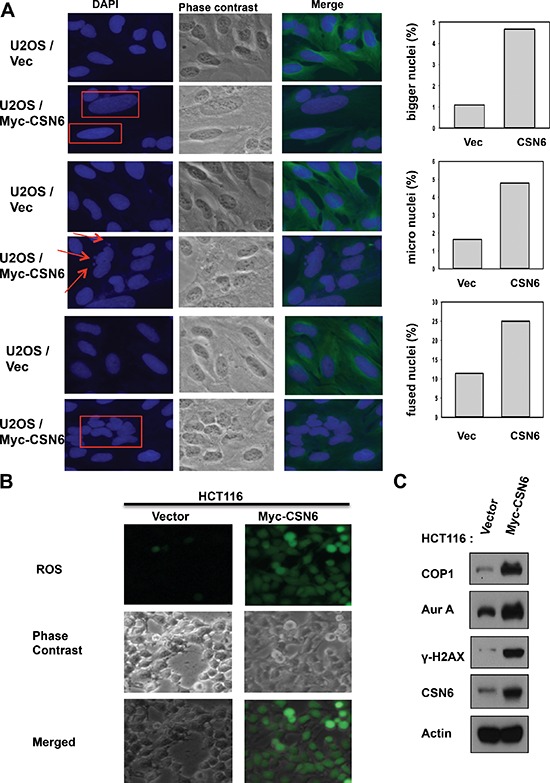
CSN6 expression leads to mitotic defect and ROS production **(A)** Stably expressing Myc-CSN6 (U2OS/Myc-CSN6) and Vector (U2OS vector) cells were stained with DAPI (4ʹ,6-Diamidino-2-Phenylindole, Dihydrochloride) and percentages of mitotic defects as demonstrated by bigger nuclei, micro nuclei, and fused nuclei were compared. Phase-contrast images and merged images of the same microscopic fields are shown. **(B)** ROS production (green fluorescence) was detected by DCFDA and fluorescence microscopy in indicated cells. Phase-contrast images and merged images of the same microscopic fields are shown. **(C)** CSN6 overexpressing cells have increased steady-state expressions of Aurk A and γ-H2AX. Equal amounts of cell lysates were immunoblotted with indicated antibodies.

CSN6 is overexpressed in many types of cancer. To understand the biological consequence of this deregulation, we established CSN6 stable expressing clones and checked their cell cycle regulation. We found that CSN6 expressing clones have increased numbers of cells with bigger nuclei, small nuclei and fused nuclei (Figure 1A), suggesting mitotic defects in these cells. We also found that these CSN6 expressing clones have elevated reactive oxygen species (ROS), suggesting potential DNA damage (Figure 1B). We then found that these cells have elevated Aurora kinase A and γH2AX (a surrogate marker of DNA double strand breaks) when compared with control cell line as assayed by immunostaining (Figure 1C). Further, these CSN6 expressing cells have high levels of COP1, a downstream target of CSN6, with concurrent high Aurora kinase A and γH2AX, as evidenced by immunoblotting. This observation suggests that CSN6-COP1 axis may be involved in DNA damage and mitotic defect.

### CSN6-mediated ROS production and DNA damage involve COP1 and Aurora A

**Figure 2:**
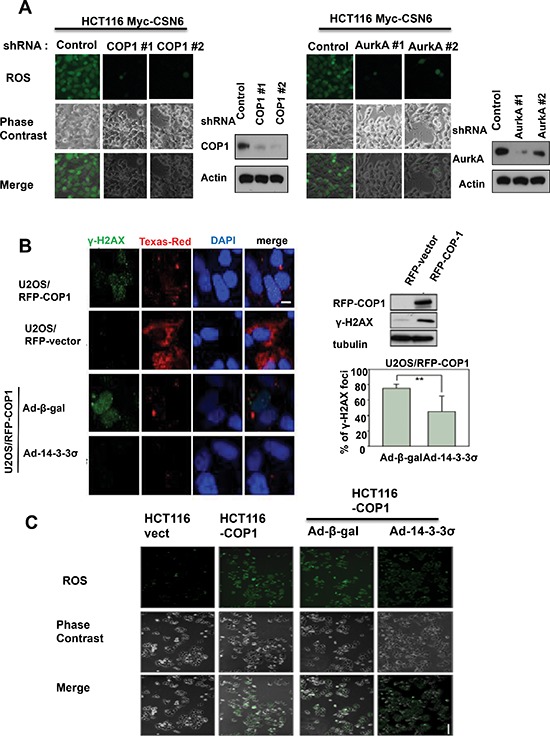
CSN6-mediated ROS production and DNA damage involve COP1 and Aurora A **(A)** Knockdown of COP1 or Aurora A attenuates CSN6-mediated ROS production. ROS production (green fluorescence) was detected by DCFDA and fluorescence microscopy in indicated cells. Phase-contrast images and merged images of the same microscopic fields are shown. **(B)** 14-3-3σ blocks the increase in γ-H2AX foci induced by COP1. Stably expressing RFP-COP1 (U2OS/RFP-COP1) and RFP vector (U2OS/RFP-vector) U2OS cells were examined for the distribution of γ-H2AX foci (green) by confocal microscope with anti-γ-H2AX and Alexa Fluor 488-conjugated secondary antibodies. DNA was counterstained with the DAPI dye (blue). Cell lysates of U2OS (RFP-vector and RFP-COP1) cells were also analyzed by immunoblotting using antibodies against γH2AX and RFP-COP1. U2OS/RFP-COP1 cells were also infected with Ad-β-gal or Ad-HA-14-3-3σ to examine their impacts on γH2AX foci formation. The bar graph shows percentage of nuclear γH2AX foci in Adenovirus-infected groups. Error bars represent 95% confidence intervals. 200 hundreds have been counted. Two asterisk, *p* < 0.01 (Student's *t*-test). Scale bar, 10 μm. **(C)** 14-3-3σ inhibits COP1-mediated ROS production. ROS production (green fluorescence) was detected by DCFDA and fluorescence microscopy in stably expressing Myc-vector HCT116 cells, Myc-COP1 HCT116 cells, Myc-COP1 HCT116 cells infected with Ad-β-gal or Ad-HA-14-3-3σ. Phase-contrast images and merged images of the same microscopic fields are shown. Scale bar, 50 μm.

To understand further the role of COP1 or Aurora A in CSN6-mediated ROS production, we knocked down COP1 or Aurora A in CSN6 expressing clones, and found that COP1 or Aurora A knockdown led to reduced ROS production in CSN6 expressing cells (Figure 2A), suggesting that COP1 or Aurora A expression is involved in CSN6's DNA damaging effect.

Since CSN6 is critical in maintaining genomic stability, we analyzed the γH2AX foci formation in cells with overexpression of COP1. We detected higher levels of γH2AX by immunoblotting and larger numbers of γH2AX foci by immunofluorescent microscopy in COP1-overexpressing cells when compared with control cells (Figure 2B), but these effects of COP1 were reversed when cells were infected with adenovirus 14-3-3σ (Ad-14-3-3σ; Figure 2B), a negative regulator of COP1 [25]. We then investigated further the impact of COP1 overexpression on ROS production by staining. Again, COP1-induced ROS production was diminished when COP1-overexpressing cells were infected with Ad-14-3-3σ (Figure 2C). These findings confirm the biological significance of the CSN6-COP-14-3-3σ-regulatory pathway in genomic stability.

### COP1 is involved in p27 protein stability regulation during DNA damage

**Figure 3:**
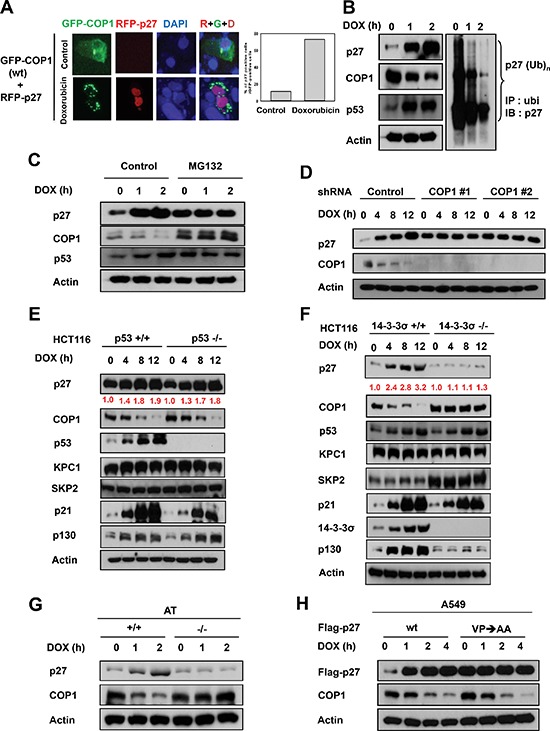
COP1 is involved in p27 protein stability regulation during DNA damage **(A)** p27 nuclear accumulation increases in response to doxorubicin. U2OS cells were treated with 1 μg/ml doxorubicin and co-transfected with either vector or GFP-COP1 and RFP-p27, stained with DAPI. **(B)** Reduced p27 ubiquitination in A549 cells after DNA damage. A549 cells were treated with 1 μg/ml doxorubicin (DOX) for the indicated times and lysates were analyzed by immunoblotting (IB) with the indicated antibodies. Cell lysates were immunoprecipitated with anti-ubi followed by immunoblotting with anti-p27. **(C)** MG132 reverses DNA damage-mediated COP1 downregulation. A549 cells treated with 1 μg/ml DOX for the indicated times were treated with MG132 for 3 hours. Lysates were analyzed by IB with the indicated antibodies. **(D)** COP1 depletion and accumulation of p27. COP1 shRNA (#1 or #2) or control shRNA HCT116 stable transfectants were treated with 1 μg/ml DOX for the indicated times. Cell lysates were analyzed by IB with the indicated antibodies. **(E)** p27 is accumulated in HCT116 p53^−/−^ cells. HCT116 p53^−/−^ cells were treated with 1 μg/ml DOX for the indicated times. Cell lysates were analyzed by IB with the indicated antibodies. **(F)** p27 is not accumulated in 14-3-3σ^−/−^ cells treated with DOX. HCT116 14-3-3σ^−/−^ cells were treated with 1 μg/ml DOX for the indicated times. Cell lysates were analyzed by IB with the indicated antibodies. **(G)** DNA damage–mediated upregulation of p27 is ATM-dependent. AT22IJE-T/pEBS7 (ATM^−/−^) or AT22IJE-T/YZ5 (ATM^+/+^) cells were treated with 1 μg/ml doxorubicin (DOX) for the indicated times. Lysates were analyzed by immunoblotting with the indicated antibodies. **(H)** p27 (VP) mutant accumulation and DNA damage. A549 cells were transfected with either wt Flag-p27 or Flag-p27 (VP→AA). Cells were treated with 1 μg/ml doxorubicin (DOX) for the indicated times. Lysates were analyzed by IB with anti-Flag, anti-COP1, or Actin.

DNA damage induces cytoplasmic distribution of COP1 and subsequent COP1 cytoplasmic ubiquitination [23–25]. The biological consequence of this regulation remains unclear. We sought to determine the relationship between the subcellular localization of COP1 and the effects of COP1 on p27 during DNA damage. Without DNA damage, COP1 is distributed in both the nucleus and the cytoplasmic compartments, and p27 expression levels were diminished when cells were co-transfected with p27 and COP1 (Figure 3A). However, we found that the p27 signal accumulated in the nucleus when COP1 was excluded in the cytoplasm (punctate green fluorescence) upon DNA damage by doxorubicin (Figure 3A), suggesting that DNA damage affects the subcellular distribution of COP1, which may in turn affect levels of p27.

In a DNA damage response study using lung adenocarcinoma A549 cells expressing wild-type p53 as a model, we found that DNA damage caused by treatment with doxorubicin led to the accumulation of p27 (Figure 3B). Levels of constitutive photomorphogenic 1 (COP1) [6, 17, 25, 41], an E3 ubiquitin ligase, was downregulated by the DNA damage at the same time (Figure 3B). Moreover, p27 ubiquitination levels were reduced after DNA damage (Figure 3B), implying that reduced ubiquitin-mediated proteasome degradation of p27 was involved in doxorubicin-mediated p27 accumulation. These observations suggest that COP1, as an E3 ubiquitin ligase, may have a functional role in DNA damage-mediated p27 accumulation. Consistently, treatment with MG132, an inhibitor of ubiquitin-mediated protein degradation via proteasomes, alleviated the impact of DNA damage on COP1 reduction or p27 accumulation (Figure 3C), suggesting the involvement of ubiquitin-mediated protein degradation in this process.

To further understand the role of COP1 in DNA damage-mediated p27 accumulation, we examined expression levels of p27 when COP1 was depleted by shRNA during DNA damage. We found that COP1 depletion led to the accumulation of p27 above the basal level at the early time points of DNA damage, as expected (Figure 3D). However, the phenomenon of p27 accumulation following DNA damage time points is no longer present when COP1 is depleted (i.e., no change following time points). We also found that accumulation of p27 after doxorubicin-induced DNA damage was observed in HCT116 p53^−/−^ cells (Figure 3E) but not in HCT116 14-3-3σ^−/−^ cells (Figure 3F), suggesting that DNA damage-mediated p27 accumulation is not p53-dependent but is 14-3-3σ-dependent. Importantly, we noted that COP1 was downregulated in HCT116 p53^−/−^ cells but not in HCT116 14-3-3σ^−/−^ cells (Figure 3E and 3F). This is because 14-3-3σ, a protein that plays a role in DNA damage [31, 42–44], was required for DNA damage-mediated downregulation of COP1 [24, 25] (Figure 3F). When 14-3-3σ is deficient, COP1 is relatively stable, thereby interfering with p27 accumulation. We also noted that DNA damage-mediated downregulation of COP1 and p27 accumulation depended on the presence of ATM, a protein collaborates with 14-3-3σ for mediating COP1 downregulation [24, 25], as DNA damage-mediated downregulation of COP1 and p27 accumulation is not observed in ATM−/− cells (Figure 3G).

COP1 preferentially binds to target proteins with the VP motifs [45]. We have analyzed the p27 peptide sequence and found a putative COP1 binding motif located in p27 (aa 87–198) [10]. We thus constructed the p27 (VP→AA) mutant, which fails to bind COP1 and is resistant to COP1-mediated ubiquitination [10]. We found that levels of p27 (VP→AA) mutant protein is pretty stable in the presence of DNA damage and is above the basal level of wt p27 at the early time points of DNA damage (Figure 3H). Importantly, the phenomenon of p27 (VP→AA) mutant protein accumulation following DNA damage time points is not observed in the presence of DNA damage (Figure 3H), suggesting that interaction between COP1 and p27 is critical for the inverse relationship during DNA damage.

### COP1 modification is required for DNA damage-mediated p27 stabilization

**Figure 4:**
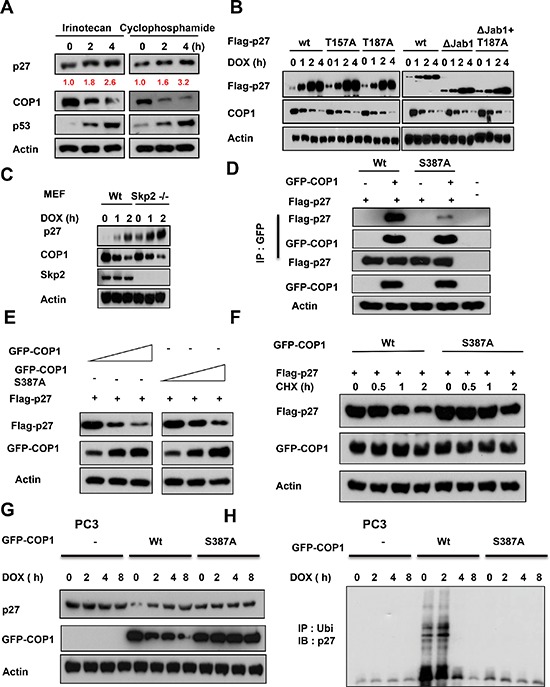
COP1 modification is required for DNA damage-mediated p27 accumulation **(A)** p27 accumulates in A549 cells after DNA damage. A549 cells were treated with 10 μg/ml irinotecan and 20 μg/ml cyclophosphamide for the indicated times and lysates were analyzed by IB with the indicated antibodies. **(B)** p27 accumulation occurs regardless of construct modifications. A549 cells were transfected as indicated with wild-type (wt) Flag-p27 or T157A, T187A, ΔJab1, or ΔJab1+T187A mutants. Cells were treated with 1 μg/ml DOX for the indicated times. Equal amounts of protein from cell lysates were analyzed by IB with anti-Flag, anti-COP1, or anti-Actin. **(C)** Skp2 is not involved in DNA damage-mediated p27 accumulation. Wild-type (wt) MEF cells and Skp2^−/−^ cells were treated with 1 μg ml doxorubicin (DOX) for the indicated times. Lysates were immunoblotted with the indicated antibodies. **(D)** COP1 S387A mutant binds to p27 with less efficiency. Cells were transfected with the indicated plasmids and equal amounts of lysates were immunoprecipitated with anti-GFP, followed by immunoblotting with indicated antibodies. **(E)** COP1 S387A mutant reduces the steady-state expression of p27 with less efficiency. Cells were transfected with the indicated plasmids and equal amounts of lysates were immunoblotted with anti-Flag to examine the expression of p27. **(F)** COP1 S387A mutant accelerates the turnover of p27 with less efficiency. Cells were transfected with the indicated plasmids and treated with CHX (100 μg/ml) for the indicated times. Cell lysates were immunoblotted with the indicated antibodies. **(G)** COP1 S387A mutant does not have inverse relationship with p27 in the presence of DNA damage. PC3 cells were transfected with the indicated plasmids and cells were treated with 1 μg/ml doxorubicin (DOX) for the indicated times. Lysates were immunoblotted with the indicated antibodies. **(H)** COP1 S387A mutant does not have impact on p27 ubiquitination in the presence of DNA damage. PC3 cells were transfected with the indicated plasmids and cells were treated with 1 μg/ml doxorubicin (DOX) for the indicated times as in (g). The cell lysates of the transfected cells from (g) were immunoprecipitated with anti-ubi and immunoblotted with an anti-p27 antibody.

We found that DNA damage-mediated accumulation of p27 is not specific to doxorubicin, as other DNA damaging agents such as irinotecan and cyclophosphamide have the same impact (Figure 4A). We next found that wild-type p27 (wt), p27 with deficient Akt phosphorylation (p27T157A), p27 that defectively binds to SKP2 (p27 T187A), and p27 missing the Jab1 binding region (p27ΔJab1; Figure 1F) [38, 46–48] all accumulated after DNA damage following concurrent downregulation of COP1, indicating that DNA damage led to p27 accumulation following COP1 downregulation regardless of modifications in the p27 construct. These observations imply that the effects of COP1 on p27 do not occur through regulating Akt, SKP2, or Jab1 (Figure 4B). We confirmed that COP1 still has inverse relationship with p27 in the presence of DNA damage in Skp2−/− cells (Figure 4C).

We have mapped COP1 S387 as the evolutionarily conserved 14-3-3σ binding motif (RTAS^387^QL) [25]. S387 is an ATM phosphorylation site, and 14-3-3σ has a role in ATM-induced COP1 nuclear exclusion. COP1 S387 phosphorylation is required for COP1–14-3–3σ binding. COP1 (S387A) mutant lost its binding affinity for 14-3-3σ; therefore, it is not regulated by 14-3-3σ or subcellular localization shuttling. We found that COP1 (S387A) binds to p27 with less efficiency as shown by co-ip experiment (Figure 4D). COP1 S387A mutant affects the steady-state expression and the turnover of p27 with less efficiency (Figure 4E). Further, COP1 S387A mutant's capability in accelerating p27 turnover is compromised when compared with wt (Figure 4F). COP1 S387A mutant does not have inverse relationship with p27 in the presence of DNA damage in PC3 cells, which does not have the expression of COP1 (Figure 4G). Accordingly, COP1 S387A mutant does not have impact on p27 ubiquitination under DNA damage in PC3 cells (Figure 4H). Together, our results show that DNA damage-mediated p27 accumulation requires ATM modification of COP1 and 14-3-3σ-binding, but does not require the function of Akt, Jab1, or Skp2.

### COP1 affects genome stability by affecting p27-Aurora A axis

**Figure 5:**
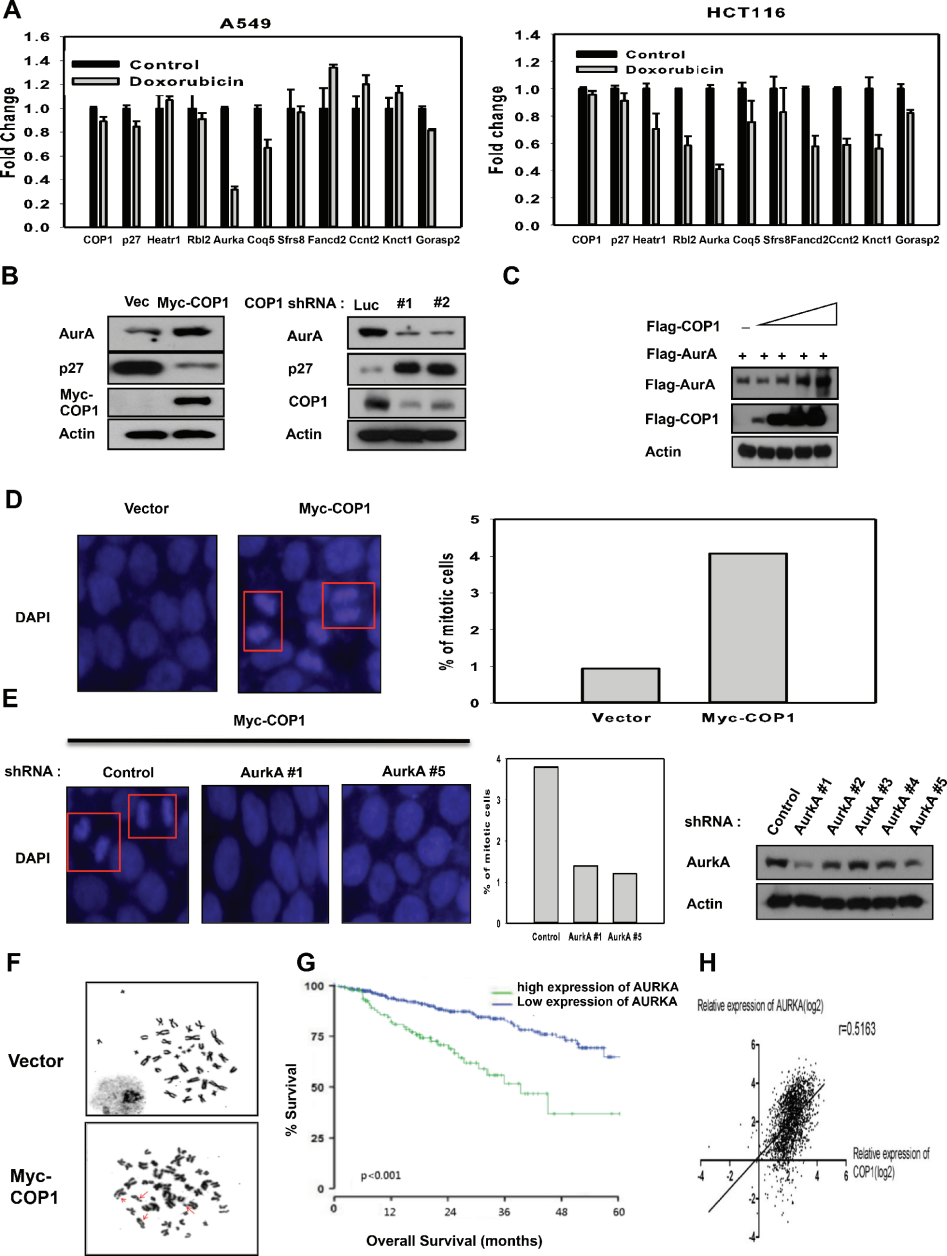
COP1 affects genome stability by affecting p27-Aurora A axis **(A)** DNA damage downregulates the expression of genes that are suppressed through p27 mediation. mRNA levels of the indicated p27 target genes were determined by quantitative reverse transcription PCR in A549 and HCT116 cells after DNA damage. **(B)** COP1 overexpression or knockdown can affect the expression of p27 or Aurora A. **(C)** COP1 upregulated Aurora A steady-state expression in a dose-dependent manner. 293T cells were co-transfected with the indicated expression vectors. Equal amounts of protein from cell lysates were analyzed by immunoblotting with the indicated antibodies. **(D–E)** Stably expressing Myc-COP1 (HCT116/Myc-COP1) and Vector (HCT116/vector) cells (d) and Myc-COP1 overexpressing HCT116 cells infected with either AurkA shRNA or control shRNA (E) were stained with DAPI (4ʹ,6-Diamidino-2-Phenylindole, Dihydrochloride) and percentage of mitotic cells were compared. Lysates of Myc-COP1 overexpressing HCT116 cells infected with either AurkA shRNA or control shRNA were immunoblotted with indicated antibodies (E, right). **(F)** COP1 overexpression leads to deregulation of genome integrity. Giemsa-stained chromosomes from metaphase-arrested cells were examined to assess genomic aberrations. Chromosomes from Myc-COP1-overexpressing HCT116 stable transfectants and vector control transfectants were shown to illustrate chromosomal fragments and chromatid breaks. **(G)** High expression of Aurora A (AURKA) correlates with poor survival of multiple myeloma patients. Kaplan-Meier curves for overall survival according to Aurora A expression in 414 patients with multiple myeloma are shown. Increased expression of Aurora A was associated with poor overall survival. **(H)** Levels of COP1 expression was positively correlated with Aurora A expression in a cohort of patients with multiple cancer data sets.

p27 plays a role in suppressing genes involved in mitosis or cell proliferation [49]. Importantly, treatment with DNA damaging agent doxorubicin led to downregulation of genes that are suppressed by p27 activation in A549 cells and HCT116 cells (Figure 5A), including Aurora A. Transfection studies showed that COP1 particularly decreased p27 expression and caused concurrent elevated expression of Aurora A, while COP1 knockdown suppressed the expression of Aurora A (Figure 5B). We found that COP1 overexpression led to elevation of Aurora A in a dose-dependent manner (Figure 5C). COP1 overexpression led to increased amount of mitotic cells (Figure 5D), while Aurora A knockdown in this COP1-overexpressing cell led to reduced number of mitotic cells (Figure 5E). Like CSN6, COP1 overexpression caused deregulation of genome integrity with increased chromosomal breakages (Figure 5F, Table 1), suggesting that the COP1-p27-Aurora A axis may play a role in genome integrity. Further, high expression of Aurora A was associated with poor overall survival (Figure 5G) in multiple myeloma, and COP1 expression was positively correlated with Aurora A expression in cancer as evident in multiple cancer data set studies (Figure 5H).

**Table 1:** COP1 expression leads to chromosome instability

	Vector	Myc-COP1
Number of metaphase examined	35	33
% normal diploid cells	97.1	45.4
% cells with chrom. aberrations	0	21.2
% cells with breaks	0	21.2
% cells with chrom. fusions	0	0

### COP1 promotes cell migration, hinders DNA damage repair, and accelerates tumor formation

**Figure 6:**
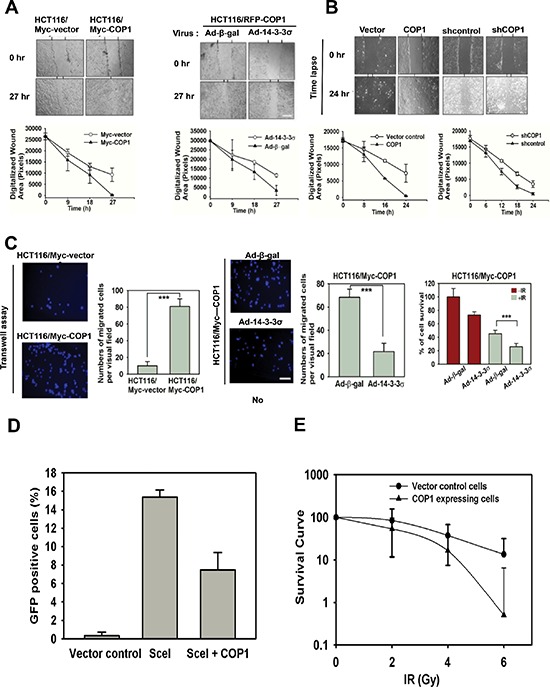
COP1 promotes cell migration, hinders DNA damage repair **(A)** COP1 promotes cell motility. Stable transfectants, HCT116/Myc-COP1 and HCT116/Myc-vector cells, were plated for wound healing assay. Stably expressing HCT116/Myc-COP1 cells were infected with Ad-β-gal or Ad-HA-14-3-3σ. Infected cells were plated for wound healing assay. Migrated cells in the wound were observed using time lapse microscopy. The size of wound healing area were digitized following the time lapse after the treatments and plotted as a line graph. Error bars represent 95% confidence intervals (*n* = 3). **(B)** COP1 knockdown reduces cell motility. U2OS cells were transfected with indicated plasmids and plated for wound healing assay. The size of wound healing area were digitized following the time lapse after the treatment and plotted as a line graph. **(C)** 14-3-3σ antagonizes COP1-promoted cell motility. HCT116/Myc-COP1 and HCT116/vector cells were plated for transwell assay. Migrated cells were stained with DAPI and counted. Stably expressing HCT116/Myc-COP1 cells were infected with Ad-β-gal or Ad-HA-14-3-3σ for 1 day. Infected cells were plated for transwell assay. Number of migrated cells was quantitated for each group and presented as a bar graph. Error bars represent 95% confidence intervals. Three asterisk, *p* < 0.001 (Student's *t*-test, *n* = 3). Scale bar, 50 μm. **(D)** COP1 overexpression reduces the repair of homologous recombination. Cells were transfected with indicated plasmids. DSB repair was shown by the percentage of cells expressing GFP using cell cycle profiles. Error bars represent 95% confidence intervals. **(E)** COP1 overexpression leads to reduced survival in response to DNA damage. Cells were irradiated with the indicated doses of IR. After 12 days, colonies were stained with 0.5% crystal violet and the colonies were counted. Surviving fractions were calculated.

CSN6 and COP1 are frequently overexpressed in cancers. On the basis of our observation, we then examined whether COP1 could affect cell migration, DNA damage repair, and tumorigenicity. COP1-expression facilitates cell migration (Figure 6A). We found that 14-3-3σ (a negative regulator of COP1) replenishment (through Adenoviral delivery) antagonized COP1-mediated cell migration (Figure 6A). Similarly, COP1 knockdown led to slower cell migration (Figure 6B). We have similar observation in transwell assay when 14-3-3σ is administrated (Figure 6C). Interestingly, COP1-overexpressing cells reduced the repair of homologous recombination, as demonstrated by DSB repair efficacy (Figure 6D). Also, COP1-overexpressing cells have reduced survival in response to DNA damage (Figure 6E), which is consistent with the fact that COP1-overexpressing cells are compromised in DSB repair.

**Figure 7:**
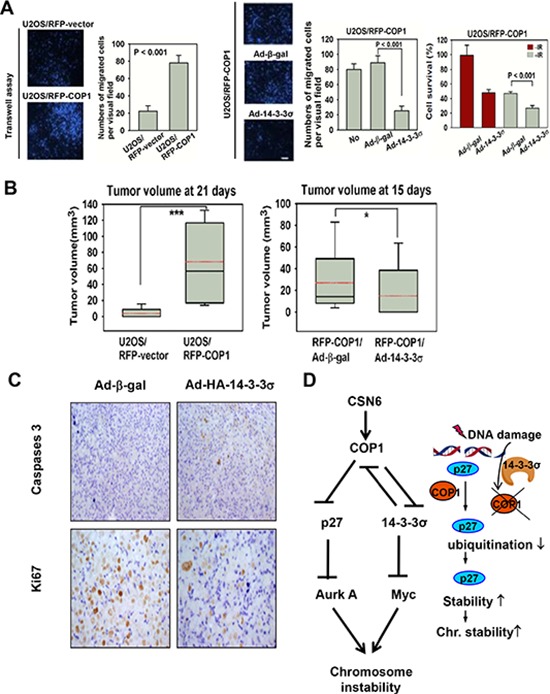
COP1 accelerates tumor formation **(A)** 14-3-3σ antagonizes COP1-mediated cell motility. U2OS/RFP-COP1 and U2OS/RFP-vector cells were plated for transwell assay. Stably expressing U2OS/RFP-COP1 cells were left uninfected, infected with Ad-β-gal or Ad-HA-14-3-3σ for 1 day. Infected cells were plated for transwell assay. Number of migrated cells was quantitated and presented as bar graphs. Error bars represent 95% confidence intervals. Three asterisk, *p* < 0.001 (Student's *t*-test, *n* = 3). Scale bar, 50 μm. **(B)** 14-3-3σ hinders COP1-mediated tumorigenicity. Indicated cells were injected subcutaneously into nude mice. Tumor volumes were measured at the indicated days. Error bars represent 95% confidence intervals. One asterisk, *p* < 0.05. Three asterisk, *p* < 0.001 (Student's *t*-test, *n* = 6). **(C)** Serial tumor sections from the experiment were stained with anti-cleaved Caspase 3 and anti-Ki67. **(D)** Model of CSN6-COP1-p27 axis in regulating chromosome stability.

To study the tumorigenicity, we set up COP1 overexpression in U2OS cells. Again, COP1-expression facilitates cell migration (Figure 7A). Also Ad-HA-14-3-3σ replenishment antagonized COP1-mediated cell migration (Figure 7A). For tumorigenicity, COP1 can promote tumorigenicity (Figure 7B) compared with control cells, while 14-3-3σ administration through Adenoviral delivery (Ad-14-3-3σ) antagonized COP1-promoted tumorigenicity (Figure 7B). In the xenografted cancer samples, 14-3-3σ administration inhibited tumor growth by increasing caspase 3 cleavage while downregulating Ki67 staining as evidenced in immunohistochemical staining (Figure 7C). These data suggest that tumors with COP1 overexpression can have growth advantage, which is a result of increased cell proliferation and reduced apoptosis.

## DISCUSSION

CSN6 expression is elevated in cancers and leads to poor survival [5, 50, 51], suggesting that abnormal CSN6 overexpression allows cancer to have many advantages. However, its role in cancer remains unclear. In the present study, we found that CSN6 deregulation is causing genome instability, one of the cancer hallmarks [52], in cancer. We found that CSN6 can link to COP1 elevation, which has unprecedented biological activity in downregulating p27, thereby inducing Aurora A; this confirms CSN6's role in promoting cancer development by influencing genome integrity.

Also, the fact that COP1 downregulation participates in reducing p27 ubiquitination/degradation under genotoxic condition has biological significance as p27 needs to be preserved in order to cause cell cycle arrest. In addition to p21, a cdk inhibitor induced by p53 to cause cell cycle arrest [31], our p27 studies show another layer of DNA damage response. Interestingly, this process of p27 induction in response to DNA damage occurs regardless of p53 status, suggesting that cells has a backup system to cause cell cycle even p53 is missing. However, this process is compromised if another tumor suppressor 14-3-3σ is silenced. We demonstrate that 14-3-3σ [42–44] is critical in keeping COP1 at bay by causing ubiquitination/degradation of COP1. Loss of 14-3-3σ leads to uncontrolled COP1 expression, thereby causing the degradation of p27. It is then conceivable that p27's pleotropic impact on genes involved in mitosis such as Aurora A kinase will be compromised. Therefore, the COP1-p27 link is translated into the regulation of genes transcriptionally suppressed by p27, including Aurora kinase A, providing explanation to COP1-mediated genome instability and a possible plethora of functions of COP1. Significantly, we have shown that COP1 expression is positively corrected with Aurora A kinase expression in human cancer samples. Thus, the COP1-p27-Aurora kinase A link can be recapitulated in cancer samples. Furthermore we have found that COP1 overexpression can hinder DSB repairs by reducing the repair of homologous recombination, as demonstrated by reduction of DSB repair efficacy (Figure 6D). Also, COP1-overexpressing cells have reduced survival in response to DNA damage, consistent with the observation that COP1-overexpressing cells are compromised in DSB repair. Additional studies investigating the way in which COP1 participates in interfering DSB repair efficacy are warranted.

In our study, 14-3-3σ inhibited COP1-mediated tumor growth in xenograft cancer mice. Tumor sample analysis indicated that 14-3-3σ ablated the COP1 activity, thereby reducing Ki67 expression levels as well as increasing apoptosis, This result could be a manifestation of reducing COP1's impact on p27 (this study) as well as p53 [41]. Because CSN6 is frequently overexpressed in cancer; therefore, COP1 stabilization and its downstream impact on p27 degradation may lead to chromosomal instability (Figure 7D). It is important to point out that COP1 is known to degrade 14-3-3σ as well [6], which in turn will lead to Myc stabilization [42], an important factor hinders DNA repair and causes chromosome instability. Thus CSN6's impact on chromosome integrity is delineated in our model (Figure 7D). Also, our results demonstrated a link between DNA damage response, COP1 regulation, p27 stability, and possible chromosome stability (Figure 7D): DNA damage response leads to p27 elevation through 14-3-3σ-mediated COP1 downregulation [24]. The role of COP1 in degrading p27 and subsequent expression of Aurora A offers a new bridge for knowledge gap regarding the role of COP1 downregulation in maintaining chromosome integrity in response to DNA damage.

## MATERIALS AND METHODS

### Cell culture and reagents

Human 293T, A549, MEF, AT22IJE-T/pEBS7 (ATM^−/−^) [53], and AT22IJE-T/YZ5 (ATM^+/+^) cells were cultured in Dulbecco's modified Eagle medium/F12 medium supplemented with 10% fetal bovine serum, 100 units/ml penicillin G, 100 μg/ml streptomycin, and 0.25 μg/ml amphotericin. Human HCT116 cells and U2OS cells were maintained as described above. Flag-p27 wt was constructed by PCR cloning and mutants (VP-AA, T157A, T187A, ΔJab1, ΔJab1 + T187A) were generated using PCR-directed mutagenesis [48]. Antibodies to the following epitopes and proteins were purchased from the indicated vendors: CSN6 (Biomol International), COP1 (Bethyl Laboratories, Inc.), γH2AX (Upstate), Aurora A (Cell Signaling Technology), ubiquitin (Zymed Laboratories), p27 (BD Transduction Laboratories and Santa Cruz Biotechnology). Flag (M2 monoclonal antibody), Actin, and Tubulin were purchased from Sigma. p53 (FL393 and DO1), KPC1, SKP2, and Myc (9E10) were purchased from Santa Cruz Biotechnology.

### Generation of stable transfectants

Cells were transfected with either PCDNA6 or PCDNA6-Myc-CSN6 plasmids and were selected in 8 mg/ml blasticidin for 2 weeks. Cells were infected by lentiviral shRNA transduction particles (Sigma, NM_006833) containing either control shRNA or CSN6 shRNA. After infection, cells were selected with 2 mg/ml puromycin for 2 weeks. For generation of RFP-tagged-COP1 (RFP-COP1) overexpression stable transfectants, U2OS cells were transfected with RFP vector or RFP-COP1 plasmids by Electroporation (Amaxa). Forty-eight hours after transfection, cells were selected in 500 μg/ml G418 containing culture medium for 4 weeks. For generation of Myc-COP1 overexpression stable transfectants, HCT116 cells were transfected with either pCDNA6 or pCDNA6-Myc-COP1 plasmids by Electroporation (Amaxa). Forty-eight hours after transfection, cells were selected in 8 μg/ml blasticidin (Invivogen) containing culture medium for 2 weeks.

### Generation of stable cell line for HR repair assay

The DR-GFP reporter substrate was integrated into cellular genomic DNA. SceGFP contains an I-SceI endonuclease site within the coding region, which abolishes GFP expression. iGFP is a truncated GFP, which contains homologous sequence for the SceGFP. Expression of I-SceI induces a single DSB in the genome. When this DSB is repaired by HR, the expression of GFP can be restored and analyzed by flow cytometry to indicate the efficiency of HR repair. Assays were performed as previously described [54].

### Immunoblotting

Total cell lysates were solubilized in lysis buffer (50 mM Tris, pH 7.5, 150 mM NaCl, 1 mM EDTA, 0.5% NP-40, 0.5% Triton X-100, 1 mM phenylmethylsulfonyl fluoride, 1 mM sodium fluoride, 5 mM sodium vanadate, 1 μg each of aprotinin, leupeptin, and pepstatin per ml) and processed as previously described [8]. Proteins were resolved by SDS-PAGE gels and then proteins were transferred (Bio-Rad) to polyvinylidene difluoride membranes (Millipore). The membranes were blocked with 5% nonfat milk for 1 h at room temperature prior to incubation with indicated primary antibodies. Subsequently membranes were washed and incubated for 1 h at room temperature with peroxidase-conjugated secondary antibodies (Thermo scientific). Following several washes, chemiluminescent images of immunodetected bands on the membranes were recorded on X-ray films using the enhanced chemiluminescence (ECL) system (Roche) according to the manufacturer's instructions.

### Immunofluorescence

RFP-COP1 expressing cells grown on chamber slides were fixed with 4% formaldehyde (Fisher). Following three 5 min washes in PBS, cells were permeabilized in 0.2% Triton-X-100 (Fisher) for 15 min. After washing, cells were blocked with 5% bovine serum albumin in PBS and then incubated for 1 h with anti-γH2AX (Upstate) at 1:4,000 dilution. Following by rinsing away the nonspecific interactions, cells were incubated with Alexa Fluor 488-anti-mouse antibody (Molecular Probes) and DAPI (Sigma) in darkness. Cells were washed three times with PBS and the coverslips were then fixed onto slides and imaged using a confocal microscope (Olympus FV300). For ROS detection, cells were treated with 2ʹ-7ʹ-dichloro fluoresin diacetate (DCFDA) (5 μg/ml, Molecular probes) and observed under fluorescent microscope as previously described [42, 55].

### Correlation between COP1 and Aurora A protein expression in cancer patients

Microarray and clinical data were obtained from the gene expression profiles of a multiple-cancer dataset of Oncomine and GEO (accession number GSE2109; https://expo.intgen.org/expo/public/).

### Cytogenetics

Metaphase chromosomes from HCT116 control and myc–COP1 transfected cells were prepared as described [42] and subjected to Giemsa staining.

### Wound healing assay

Cells were cultured in medium containing 10% FBS were seeded into wells of 24-multiwell plates. After the cells grew to confluence, cells were wounded by scratching with a 10 μl sterile pipette tips. Cells were observed under time-lapse microscope every 2 h (Zeiss Axiovert 200 M microscope). Wounded area was defined by line. Cells growing into lines are considered as wound closure.

### Cell motility assay

Cell motility assays were determined using 8 μm pore size, 6.5-mm polycarbonate transwell filters (Corning Coaster Corp.). 5 × 10^4^ cells were seeded in the upper chamber in 0.1% FBS medium. McCoy's 5A medium with 10% FBS was added in the bottom well to serve as chemoattractant. Cells were cultured for 8 h. Cells on the inside of the transwell inserts were removed with a cotton swab, and cells on the underside of the inserts were fixed with 4% formaldehyde, stained with 5 μg/ml DAPI, and counted using a Olympus IX70 fluorescent microscope. Each experiment was done in triplicate.

### Nude mice experiment

Four- to 6-week-old nude mice (Charles River Laboratories) were maintained in the animal facility at the University of Texas M. D. Anderson Cancer Center. Mice were divided into experimental groups, five for each. Groups included vector control stable cell lines, COP1 expressing cells left uninfected, COP1 expressing cells infected with Ad-β-gal (MOI = 10) or Ad-14-3-3σ (MOI = 100) for forth-eight hours [34]. Cells were harvested and injected into the each flank of mice. Tumor volumes were measured and recorded three times a week from day 10 after cell inoculation. At the end of experiment, the mice were sacrificed, and the tumors were removed and weighed.
